# Effects of Toasting Time on Digestive Hydrolysis of Soluble and Insoluble 00-Rapeseed Meal Proteins

**DOI:** 10.1007/s11746-017-2960-8

**Published:** 2017-02-11

**Authors:** Sergio Salazar-Villanea, Erik M. A. M. Bruininx, Harry Gruppen, Patrick Carré, Alain Quinsac, Antonius F. B. van der Poel

**Affiliations:** 1 Wageningen Livestock Research, Wageningen, The Netherlands; 2 Animal Nutrition Group, Wageningen University and Research, P.O. Box 338, 6700 AH Wageningen, The Netherlands; 3 Agrifirm Innovation Center BV, Royal Dutch Agrifirm Group, Apeldoorn, The Netherlands; 4 Laboratory of Food Chemistry, Wageningen University and Research, Wageningen, The Netherlands; 5 CREOL/OLEAD, Pessac, France; 6 Terres Inovia, Paris, France

**Keywords:** Hydrolysis rate, Maillard, Protein solubility, Rapeseed meal, Toasting

## Abstract

Thermal damage to proteins can reduce their nutritional value. The effects of toasting time on the kinetics of hydrolysis, the resulting molecular weight distribution of 00-rapeseed meal (RSM) and the soluble and insoluble protein fractions separated from the RSM were studied. Hydrolysis was performed with pancreatic proteases to represent *in vitro* protein digestibility. Increasing the toasting time of RSM linearly decreased the rate of protein hydrolysis of RSM and the insoluble protein fractions. The extent of hydrolysis was, on average, 44% higher for the insoluble compared with the soluble protein fraction. In contrast, the rate of protein hydrolysis of the soluble protein fraction was 3–9-fold higher than that of the insoluble protein fraction. The rate of hydrolysis of the insoluble protein fraction linearly decreased by more than 60% when comparing the untoasted to the 120 min toasted RSM. Increasing the toasting time elicited the formation of Maillard reaction products (furosine, *N* ^ε^-carboxymethyl-lysine and *N* ^ε^-carboxyethyl-lysine) and disulfide bonds in the insoluble protein fraction, which is proposed to explain the reduction in the hydrolysis rate of this fraction. Overall, longer toasting times increased the size of the peptides resulting after hydrolysis of the RSM and the insoluble protein fraction. The hydrolysis kinetics of the soluble and insoluble protein fractions and the proportion of soluble:insoluble proteins in the RSM explain the reduction in the rate of protein hydrolysis observed in the RSM with increasing toasting time.

## Introduction

The production of defatted 00-rapeseed meal (RSM) involves toasting for the removal of remnant solvent after oil extraction, the inactivation of myrosinase and the degradation of glucosinolates [1–3], the main antinutritional factors of RSM for monogastric animals. Increasing the toasting time decreases protein solubility [1, 3] and the contents of lysine [1, 3, 4] and reactive lysine [2, 3] of the resulting RSM. Reactive lysine can be considered as the protein-bound lysine with a free ε-amino group [5]. Nutritionally, the changes in protein solubility, lysine and reactive lysine contents have been noticed as reduction of *in vitro* crude protein digestibility [2], apparent ileal protein digestibility in broilers [6], standardized ileal protein digestibility in pigs [2] and apparent total tract crude protein digestibility in rats [1] and pigs [7].

Chemical and physical modifications can impair the accessibility of proteins for enzymatic hydrolysis [8]. The proteases in the pancreatic secretions at the small intestine are highly specific. Trypsin cleaves peptide bonds involving the carboxyl groups of either lysine or arginine [9], which are also the most susceptible amino acids to heat damage. Lysine and arginine residues that are modified via Maillard reactions could, therefore, reduce enzyme accessibility for proteolysis, finally reducing their standardized ileal digestibility [5]. Also, physical modifications, such as protein aggregation, might have a similar effect. Protein aggregates can be formed after hydrothermal processing and are noticed as a reduction in protein solubility [10]. The formation of aggregates can reduce protein accessibility for enzymatic hydrolysis [11].

In proteins that are accessible for enzymatic hydrolysis, the gastric and intestinal enzymes cleave proteins into peptides, followed by cleavage into free small peptides and free amino acids by the brush border enzymes in the gut’s epithelium. The size of the peptides produced during hydrolysis depends on the accessibility of the protein for the enzymes. Even at similar degrees of protein hydrolysis, molecular size distribution of the resulting peptides can be different [12]. This might be the result of the selectivity of the enzymes or the accessibility of the proteins for cleavage [12].

The aim of the present study was to determine the effects of toasting time during the production process of RSM on the kinetics of hydrolysis of proteins present in the complete material, and in its soluble and insoluble fractions, and on the resulting molecular size distribution of the peptides after hydrolysis. Previous results from our research group indicate that there is a highly significant positive correlation between nitrogen solubility and the rate of protein hydrolysis [3]. Therefore, we hypothesize that the rate of hydrolysis will be higher for the soluble protein fraction compared with the insoluble protein fraction, but that the rates at different toasting times will not vary within the soluble and the insoluble protein fractions. We also hypothesize that damage to the proteins due to prolonged toasting times will change the molecular size distribution of the peptides obtained after hydrolysis towards a larger size.

## Materials and Methods

### Materials

Rapeseed meals were prepared from 00-rapeseed (*Brassica napus*) at the pilot plant of CREOL/OLEAD (Pessac, France). Trypsin (type IX-S, 13,000–20,000 BAEE units/mg protein, EC 232-650-8), chymotrypsin (type II, ≥40 units/mg protein, EC 232-671-2) and peptidase from porcine intestinal mucosa (50–100 units/g solid, EC 232-875-1) were obtained from Sigma-Aldrich (St. Louis, MO, USA). The furosine, lysinoalanine and *N* ^ε^-carboxymethyl-lysine standards were obtained from PolyPeptide Laboratories (Strasbourg, France), whereas the rest of the standards (^13^C_6_,^15^N_2_-lysine, lysine, lanthionine) were obtained from Sigma-Aldrich (Steinheim, Germany).

### Rapeseed Meals Preparation

An untoasted RSM was prepared by cold-pressing of the 00-rapeseeds, solvent-extraction and desolventization using indirect heat. The 00-rapeseeds were cold-pressed (La Mecanique Moderne MBU 75 type, Arras, France) at 250 kg/h with temperatures not exceeding 80 °C. Solvent extraction was performed at temperatures not higher than 55 °C on a belt extractor (B-1930, Desmet-Ballestra, Zaventem, Belgium) at 230 l/h flow of hexane and 160 kg/h flow of the rapeseed cake. Desolventization with indirect heat (without direct steam) was performed in a desolventizer toaster (Schumacher type, Desmet-Ballestra) for 60 min at temperatures of 90 ± 3 °C.

**Fig. 1:**
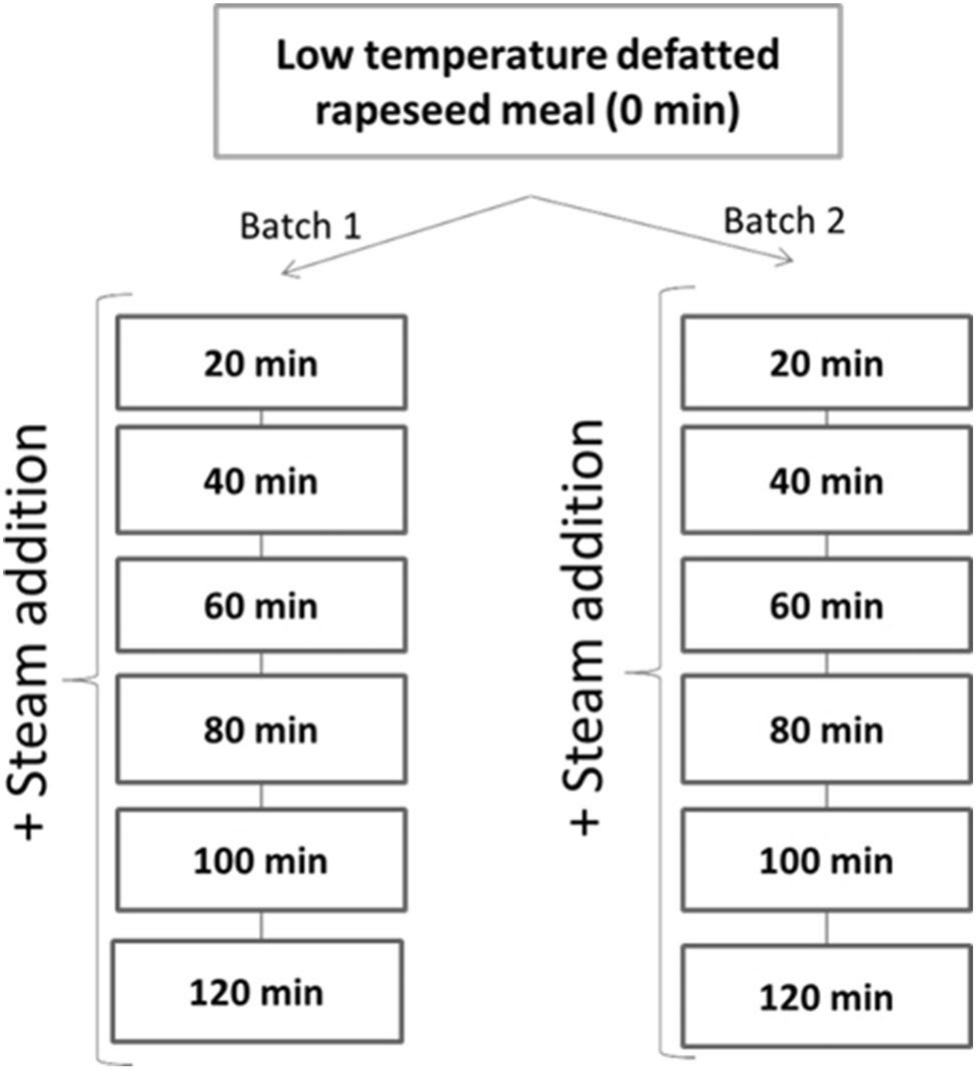
Schematic view of the design of the experiment

A batch of 150 kg of the untoasted RSM was toasted with the use of direct steam (30 kg/h) for 120 min, with spot samples of 5 kg taken every 20 min (Fig. 1). Toasting of a separate batch of 150 kg of RSM was performed during the next day under the same conditions for replication. Temperatures during toasting on the first day ranged from 107 to 112 °C and between 109 and 112 °C on the second day. In total, 13 samples of RSM (1 untoasted RSM and 12 toasted RSM) were obtained. Untoasted and toasted RSM were ground with a centrifugal mill (ZM200, Retsch, Haan, Germany) at 8000 rpm to pass a 1-mm sieve.

### Fractionation of Proteins into Soluble and Insoluble Fractions

The water-soluble and water-insoluble fractions of the meals were separated by suspending 25 g of the RSM in 250 ml of water. The pH of the suspension was adjusted to 8.0 with NaOH and magnetic stirring was applied for 20 min at room temperature. The soluble fraction was separated by centrifugation (11,900×*g*, 20 min, room temperature). Transparent solutions were obtained, which were dialyzed extensively against a 0.01 M NaCl solution. After dialysis, the pH was re-adjusted to 8.0 with NaOH. The soluble fractions were kept at 4 °C and hydrolyzed within 24 h. The insoluble fraction was filtered through a nylon cloth and washed three times with 250 ml of water in order to remove soluble proteins and was freeze-dried prior to hydrolysis.

### Analytical Methods

Nitrogen contents of the complete meals and the insoluble fractions were determined by combustion (AOAC 968.06, Thermo Quest NA 2100 Nitrogen and Protein Analyzer, Breda, The Netherlands). The N concentration of the soluble protein fractions was measured after an aliquot of 0.3 ml was oven-dried at 60 °C overnight.

### Protein Hydrolysis

*In vitro* protein hydrolysis was performed for 120 min using a modification of the method described previously [13]. Briefly, 10 ml of aqueous suspension containing 1 mg N/ml was adjusted to pH 8.0 in a titration unit (719 S Titrino, Metrohm, Herisau, Switzerland) at 39 °C using 0.1 M NaOH. At this point, 1 ml of enzyme solution containing 1.61 mg trypsin, 3.96 mg chymotrypsin and 1.18 mg porcine intestinal peptidase was added and the 120 min titration was initiated. This procedure was used for the complete RSM and the insoluble fractions.

Concerning the soluble fraction, different concentrations of N in the soluble fractions were obtained upon solubilizing the RSM in water. Demineralized water was used for diluting these solutions to the concentration of the lowest one of the solutions (0.30 mg N/ml), to a volume of 10 ml. The solutions were adjusted to pH 8.0 in the titration unit using 0.05 M NaOH. At this point, 1 ml of an enzyme solution containing 0.48 mg of trypsin, 1.19 mg of chymotrypsin and 0.35 mg of porcine intestinal peptidase was added and the 120 min titration initiated. The same substrate to enzyme ratio was used for hydrolysis of RSM, soluble and insoluble fractions. All hydrolyses were performed in duplicate.

The volume of alkali added was used for the calculation of the degree of hydrolysis according to Eq. 1,

1 $${\text{DH}} (\% ) = \frac{{{\text{Vb}} \times {\text{Nb}}}}{{\alpha \times {\text{mp}} \times {\text{htot}}}}{ \times } 100$$

in which Vb is the volume added (ml), Nb is the normality of the titration solution, α is the degree of dissociation of the α-NH_2_ group (in this case, 0.794 at 37 °C and pH 8.0), mp is the mass of protein (g) and htot is the total number of peptide bonds per gram of protein (7.8 meq/g) [14].

Modelling of the degree of hydrolysis curves was based on second-order reaction kinetics, which is described in Eq. 2,

2 $${\text{DH}} (\% ) = {\text{DH}}_{ \text{max} } - \frac{{{\text{DH}}_{ \text{max} } }}{{1 + k \times t \times {\text{DH}}_{ \text{max} } }}$$

in which DH_max_ is the maximum degree of hydrolysis (%), *k* is the hydrolysis rate constant (/M×s) and *t* is the hydrolysis time. Fitting of this model was performed using the MODEL procedure in SAS [15].

### Nitrogen Solubility

Five hundred mg of RSM were suspended in 10 ml of water and the suspension was adjusted to pH 8.0 using 2 M NaOH. The suspension was stirred for 20 min at room temperature and centrifuged (16,100×*g*, 15 min, room temperature). An aliquot (0.3 ml) was oven-dried overnight at 60 °C and analyzed for N content. Nitrogen solubility of the insoluble protein fractions were determined in water, 100 mM sodium phosphate buffer pH 7.5, and the same buffer containing either 2% (w/v) SDS, 10 mM dithiothreitol (DTT) or both 2% (w/v) SDS and 10 mM DTT. Briefly, 1.5 ml of these solutions were added to 75 mg of the insoluble protein fractions. The suspensions were vortexed for 20 s and mixed in a head-over-tail rotator for 20 min at 20 rpm. Following centrifugation (16,100×*g*, 15 min, room temperature), 0.3 ml of the supernatant was oven-dried overnight at 60 °C and analyzed for N content.

### Size Exclusion Chromatography (SEC)

After hydrolysis, a sample of 1.5 ml was taken from the supernatant of the hydrolysate, heated at 99 °C for 15 min and centrifuged (16,100×*g*, 10 min, room temperature). In addition, samples from the water-soluble fraction of RSM were analyzed after centrifugation (16,100×g, 15 min, room temperature). The samples were analyzed in the ÄKTA micro system (GE Healthcare, Uppsala, Sweden) using a Superdex 75 column (GE Healthcare) at a flow rate of 100 μl/min with UV detection at 220 nm. The eluent used was 10 mM sodium phosphate buffer of pH 7.0 containing 150 mM NaCl and 2% (w/v) SDS. The volume of injection was 50 μl. A calibration curve of the elution volumes in the column was obtained using threonine (119 Da), proline-glycine-glycine (229 Da), vitamin B12 (1355 Da), lysozyme (14,307 Da), β-lactoglobulin (18,400 Da), and ovalbumin (42,700 Da). Areas under the curve were integrated manually and the proportions of peptides based on AU response in each region (>10, 10–1.5 and <1.5 kDa) were calculated relative to the total area under the curve.

### Maillard Reaction Products, Crosslinked Compounds and Lysine

The contents of furosine, *N* ^ε^-[carboxymethyl]-lysine, lysinoalanine, lanthionine, *N* ^ε^-[carboxyethyl]-lysine and lysine in the RSM and the insoluble protein fractions were quantified by UHPLC-MS. The samples (10 mg) were hydrolyzed with 1 ml of 6 M HCl during 24 h at 110 °C. The tubes were dried under N_2_ flow and the dried material was re-suspended in 1 ml of UPLC-grade Milli-Q water, sonicated and centrifuged (16,100×*g*, 3.5 min, room temperature). The supernatant was diluted 50 times in eluent A that contained 1 mg/l (w/v) ^13^C_6_^15^N_2_-lysine (Sigma-Aldrich, Steinheim, Germany) as internal standard. Eluent A was UPLC-grade Millipore water containing 0.1% (v/v) formic acid and eluent B was acetonitrile containing 0.1% (v/v) formic acid. The samples were analyzed using an Accela RP-UHPLC system (Thermo Scientific, San Jose, CA, USA) with an Acquity BEH Amide Vanguard precolumn (2.1 × 50 mm, 1.7 μm particle size) and an Acquity UPLC BEH 300 Amide column (2.1 × 150 mm, 1.7 μm particle size). The column was maintained at 35 °C and the injection volume was 1 μl. The elution profile was as follows: 0–2 min isocratic on 80% B, 2–3 min linear gradient from 80% B to 65% B, 3–5 min isocratic on 65% B, 5–7 min linear gradient from 65% B to 40% B, 7–10 min isocratic on 40% B, 10–12 min linear gradient from 40% B to 80% B and 12–28 min isocratic on 80% B. The flow rate was 350 μl/min. Mass spectrometric data were obtained using a LTQ-VelosPro (Thermo Scientific) equipped with a heated electrospray ionization (ESI) probe, coupled to the UHPLC system. The capillary voltage was set to 3 kV. The sheath gas flow rate was set at 20 and the auxiliary gas flow rate at 5 (arbitrary units). A selected reaction monitoring (SRM) method (Table 1) was used for fragments analysis in negative ion mode for lysinoalanine and in positive ion mode for the other compounds. The normalized collision energy was set at 30 for furosine, lysine and lysinoalanine and at 35 for the other compounds, and the m/z width on the fragment was set to 1. An external standard calibration curve for furosine, lysine, lysinoalanine, lanthionine, *N* ^ε^-carboxymethyl-lysine and *N* ^ε^-carboxyethyl-lysine with concentrations of 0.01, 0.1, 1, 2.5, 5 and 10 mg/l of each standard was used to calculate the content of each compound. Compounds were quantified using the external standard calibration curve by plotting the MS peak area divided by the MS peak area of the labelled Lys, used as internal standard. Data were acquired and analyzed using XCalibur 2.2 software (Thermo Scientific).

**Table 1:** Selected reaction monitoring conditions

Compound	Parent mass^a^ (Da)	Fragment (*m*/*z*)
Lysine	146	130
^13^C_6_^15^N_2_-Lysine	154	137
*N* ^ε^-Carboxymethyl-lysine	204	84, 130
Lanthionine	208	120
*N* ^ε^-Carboxyethyl-lysine	218	84, 130
Lysinoalanine	233	128, 145
Furosine	255	84, 130

^a^Parent mass is defined as the molecular mass of the compounds before ionization

### Statistical Analysis

Linear and quadratic regressions were fitted using toasting time as fixed effect in the model. Linear and quadratic effects were considered to be significant if the *P* value was lower than 0.05 and as trends when the *P* value was between 0.05 and 0.10. Correlations between hydrolysis parameters and molecular size distribution after hydrolysis were performed using the CORR procedure of SAS [15].

## Results and Discussion

There were linear (*P* < 0.001) and quadratic (*P* = 0.001) effects of toasting time on N solubility of the RSM in water at pH 8.0 (Table 2). Solubility seems to decrease faster at shorter toasting times compared with longer ones. The decrease in N solubility can be caused by physical aggregation of proteins following protein unfolding [10]. In addition, chemical modifications to proteins (e.g. formation of intermolecular disulfide bonds, Maillard reactions) might also be involved in the solubility decrease [10]. Both of these phenomena (physical aggregation and chemical modifications) might decrease the accessibility of proteins for enzymatic hydrolysis.

**Table 2:** N solubility at pH 8.0 of the RSM and kinetic parameters for the hydrolysis curves of RSM toasted for different times and the soluble and insoluble fractions separated from these RSM

Toasting time	N solubility RSM	RSM		Soluble protein fraction		Insoluble protein fraction	
	(% of total N)	DH_max_ (%)	*k* (/M×s)	DH_max_ (%)	*k* (/M×s)	DH_max_ (%)	*k* (/M×s)
0 min	31.3	20.0	6.8E−05	14.7	1.2E−04	19.5	4.0E−05
20 min	21.1	18.3	8.0E−05	14.8	1.4E−04	22.8	4.0E−05
40 min	17.7	19.2	7.0E−05	15.3	1.3E−04	21.1	4.3E−05
60 min	15.3	19.0	6.7E−05	14.3	1.5E−04	21.4	2.4E−05
80 min	12.3	20.4	5.0E−05	15.8	1.6E−04	21.9	2.2E−05
100 min	11.4	20.2	4.8E−05	14.5	1.7E−04	21.7	2.4E−05
120 min	9.9	21.7	3.4E−05	15.3	1.4E−04	22.3	1.5E−05
SEM	0.2	0.3	4.5E−06	0.2	6.7E−06	0.3	3.0E−06
*P* value							
Linear	<0.001	0.006^b^	<0.001^c^	0.47	0.18	0.34	<0.001^d^
Quadratic	0.001^a^	0.06	0.07	0.96	0.34	0.86	0.87

*DH*_*max*_ maximum degree of hydrolysis, *k* rate of protein hydrolysis, *RSM* rapeseed meal, *SEM* standard error of the mean

^a^N solubility = 28.84 − 0.33 × time + 0.0015 × time^2^ (*R* ^2^ = 0.95)

^b^RSM DH_max_ = 18.33 + 0.023 × time (*R* ^2^ = 0.52)

^c^RSM *k* = 8.30E−05 − 3.71E−07 × time (*R* ^2^ = 0.80)

^d^Insolubles *k* = 4.46E−05 − 2.40E−07 × time (*R* ^2^ = 0.78)

### Hydrolysis Kinetics

**Fig. 2:**
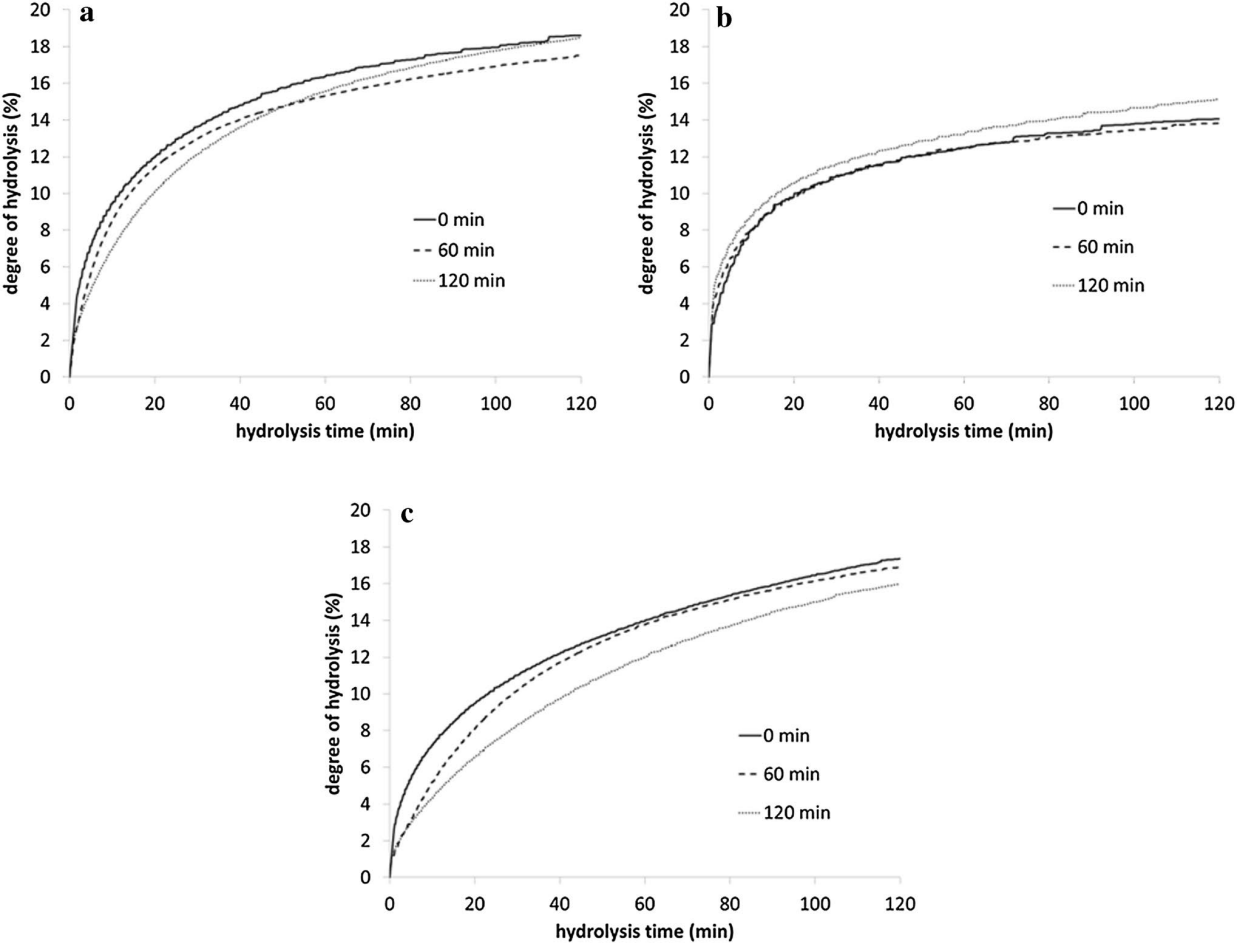
Degree of hydrolysis during 120 min hydrolysis of **a** rapeseed meals toasted for different times and **b** soluble and **c** insoluble protein fractions separated from these rapeseed meals

Increasing the toasting time affected the hydrolysis profile of RSM (Fig. 2a). The hydrolysis rate constant (*k*) of the RSM decreased linearly (*P* < 0.001) with increasing toasting time (Table 2). The *k* after toasting for 120 min was approximately 2-fold lower compared with the *k* of the untoasted RSM (6.8E−05 vs. 3.4E−05 /M×s, respectively). The decrease in *k* was probably related to the restricted enzyme accessibility for proteolysis due to protein aggregation or chemical protein modifications [8]. Increasing the toasting time caused a linear (*P* = 0.006) increase in the DH_max_ of RSM (Table 2). The DH_max_ decreased 9% after the initial 20 min of toasting and subsequently gradually increased with increasing toasting time, up to a level in the 120 min-toasted RSM similar to the untoasted RSM (Fig. 2a).

Toasting time had no effect on the *k* or the DH_max_ of the soluble protein fraction (Table 2), as can be seen from the similar shapes of the degree of hydrolysis curves (Fig. 2b). The soluble proteins could either be native or aggregated proteins with a molecular size that allows them to stay in solution [10, 16]. Aggregation of proteins in solution into spherical particles occurs when heating is performed at a pH close to the isoelectric point of the protein [17]. With longer heating times, there is secondary aggregation of these spherical particles [17] into protein condensates [16], which results in the reduction of protein solubility. Protein aggregation mechanisms for proteins in solution have been clearly identified before [16, 17]. We assume that proteins thermally treated under semi-dry conditions (e.g. toasting or autoclaving) follow similar aggregation mechanisms as reported for proteins in solution.

The *k* of the soluble protein fraction was 3–9-fold greater compared with that of the insoluble protein fraction (Table 2), which matches our hypothesis. Higher rates of hydrolysis were reported previously [18] for soluble sodium caseinate compared with the insoluble form of the same ingredient (casein), although these protein sources were not heat-processed before hydrolysis. Both native and denatured proteins can remain in solution and this depends on their concentration and on their extent of aggregation [19, 20]. Due to their high flexibility, native proteins in solution are in dynamic equilibrium with their distorted forms and these distorted forms could be considered as denatured [19], which could make them more accessible for enzymatic cleavage. Furthermore, the relatively small size of the protein aggregates present in the soluble fraction allows them to stay in solution [10]. Therefore, the lower degree of aggregation of the proteins in the soluble fraction as compared with the proteins in the insoluble fraction probably facilitated enzymatic hydrolysis, thus increasing *k*. This would mean that a decrease in the proportion of soluble to insoluble proteins after thermal processing leads to a decrease in the *k* for the complete material, as observed for the RSM in this study. Another possibility for the differences in *k* between the soluble and insoluble fractions is that the insoluble fiber matrix structures present in the latter, and not in the soluble fraction, might limit and decrease the rate of protein hydrolysis.

The *k* of the insoluble protein fraction decreased linearly (*P* < 0.001) by 62% when comparing the untoasted RSM to the 120 min-toasted RSM (Table 2), whereas no effects were noticed on the DH_max_ of these fractions. As 70–90% of the proteins in RSM correspond to insoluble proteins, the pattern of the hydrolysis curves of the insoluble protein fractions (Fig. 2c) were, as expected, similar to those from the RSM. It is possible that there is an increase in the size of protein aggregates formed at increasing toasting times, which can hamper the penetration of the hydrolytic enzymes.

*In vivo*, enzymatic protein digestion starts at the stomach with pepsin and is followed by secretion of trypsin and chymotrypsin at the small intestine, where most of the digestion occurs. The length of the small intestine and the transit time, however, are limited and the extent of protein digestion probably also depends on their rate of digestion. These factors might be even more important for poultry than for pigs, due to the short digestive tract of the former [21].

### Nitrogen Solubility of the Insoluble Fractions

**Fig. 3:**
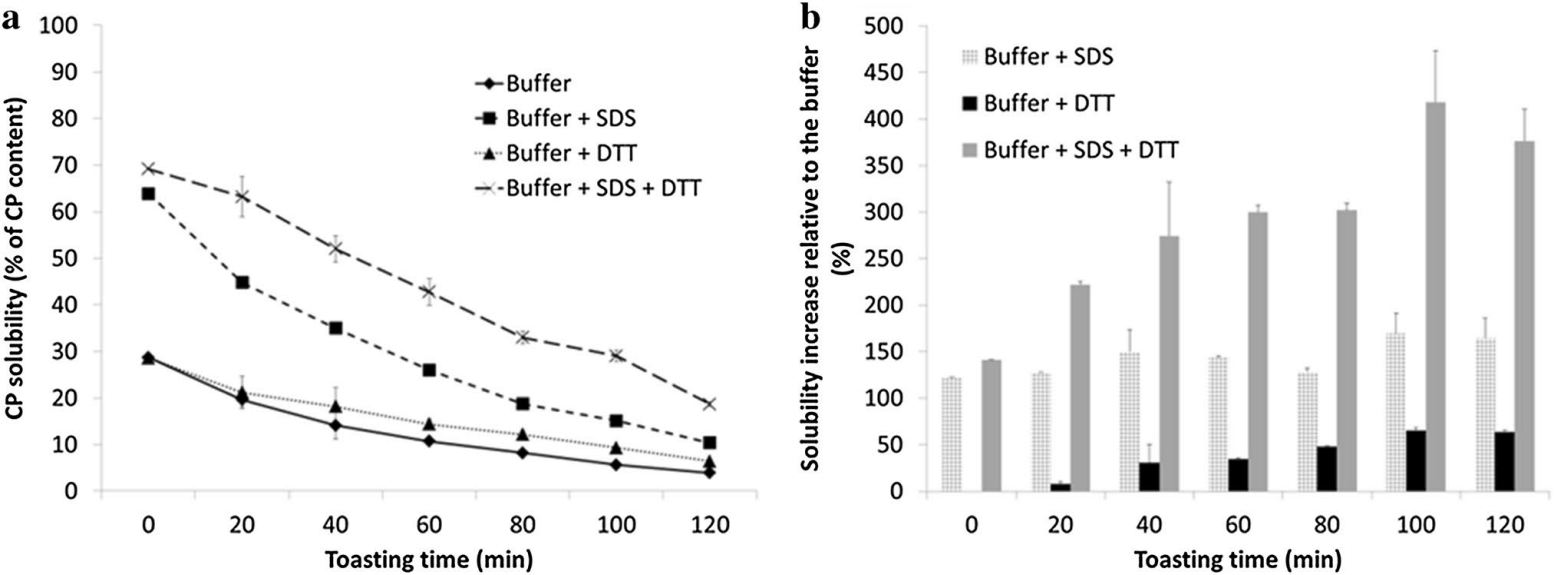
**a** Nitrogen solubility of the insoluble protein fractions in 100 mM phosphate buffer and the buffer containing either 2% SDS, 10 mM DTT or 2% SDS and 10 mM DTT. **b** Increase in N solubility of the insoluble protein fractions relative to the phosphate buffer of solutions containing either 2% SDS, 10 mM DTT, or 2% SDS and 10 mM DTT

The N solubilities of the insoluble protein fraction in the sodium phosphate buffer pH 7.5, buffer with 2% (w/v) SDS, buffer with 10 mM DTT and buffer with 2% (w/v) SDS and 10 mM DTT decreased with increasing toasting time (Fig. 3a). All solvents show a reduction in their solubilizing power with increasing toasting time, which might be related to the increase in chemical modifications of the residues (e.g. Maillard reactions or crosslinking). Non-covalent electrostatic interactions can be cleaved by salt solutions [22], such as the phosphate buffer used in this experiment. Furthermore, SDS can cleave hydrogen bonds and hydrophobic interactions, whereas DTT can cleave disulfide bonds [22]. Non-covalent bonds may be important for the stability of the insoluble aggregates. The SDS solution solubilized twice the amount of N than the phosphate buffer alone, while the increase of N solubility with additional DTT was minimal (Fig. 3a). The increase in N solubility relative to the solubility by the phosphate buffer is reported in Fig. 3b. The relative importance of non-covalent bonds (solubilized by SDS solution) for the stability of the aggregates does not change with the increasing toasting time (Fig. 3b). With increasing toasting time, there is an increase in the relative amount of protein solubilized by the DTT-containing buffer (Fig. 3b). This indicates that there is formation of disulfide bonds in the insoluble aggregates with increasing toasting time. The SDS–DTT solution solubilized more protein than SDS or DTT containing solutions separately. Synergy of SDS and DTT for N solubility has been reported before [23] for extruded soy proteins. In addition, the relative amount of protein solubilized by this solution increased with increasing toasting time (Fig. 3b). We suggest that cleavage of non-covalent bonds by SDS exposes extra disulfide bonds that can subsequently be cleaved by the DTT.

### Maillard Reaction Products

**Fig. 4:**
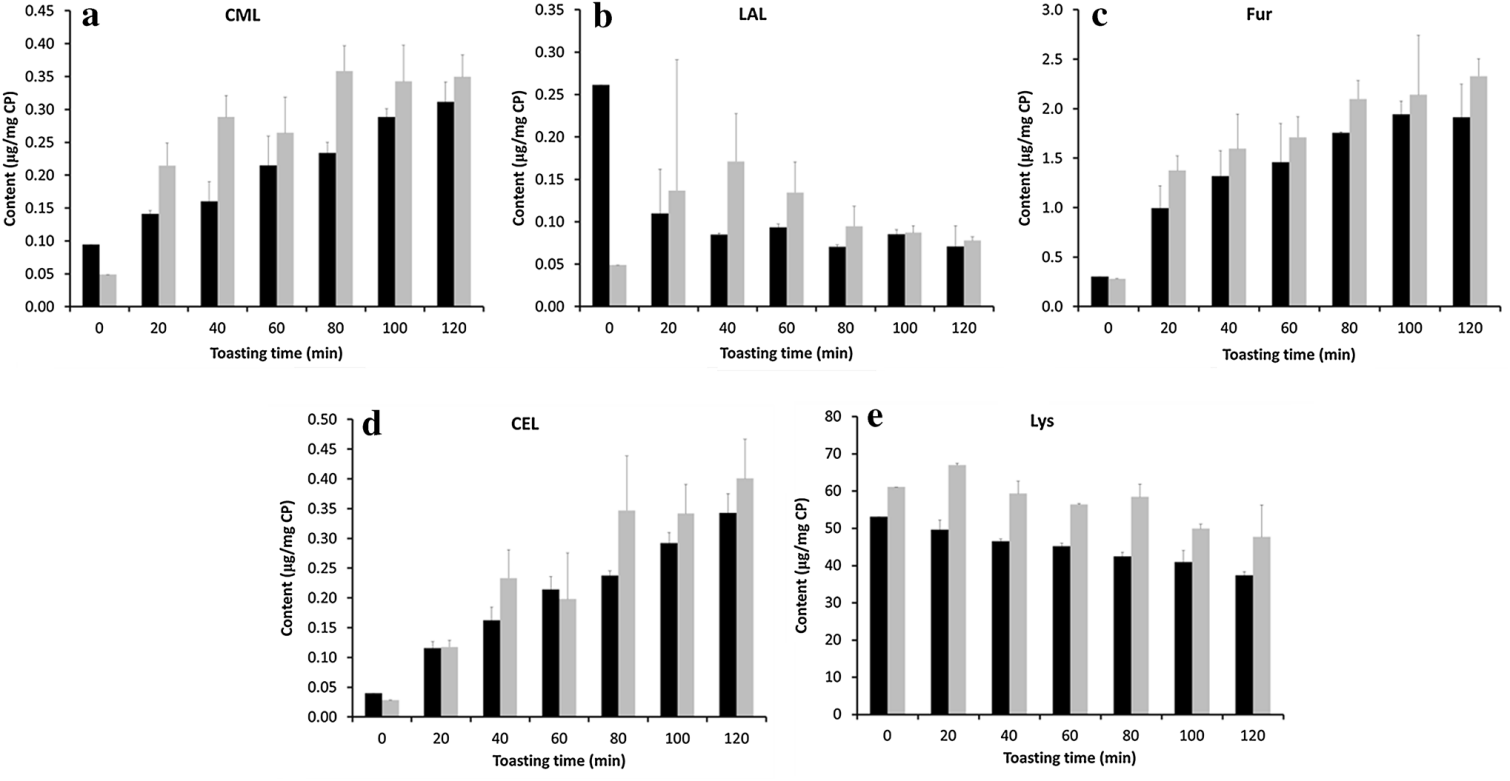
**a** *N* ^ε^-[carboxymethyl]-lysine (CML), **b** lysinoalanine (LAL), **c** furosine (Fur), **d** *N* ^ε^-[carboxyethyl]-lysine (CEL) and **e** lysine (Lys) contents (μg/mg crude protein) in the rapeseed meals (*black*) toasted for different times and the insoluble protein fraction (*gray*) separated from these rapeseed meals

Formation of early (fructoselysine) and advanced Maillard reaction products (*N* ^ε^-carboxymethyl-lysine and *N* ^ε^-carboxyethyl-lysine) in the RSM was noticed with increasing toasting time, along with a decrease in the lysine content (Fig. 4). The lysine contents in the present study (<5.3 g/100 g CP) were lower than those reported previously [4] for untoasted (6.0 g/100 g CP) and commercially toasted (5.6 g/100 g CP) canola meals. The decrease in lysine content (7%) reported in that study when comparing the untoasted to the toasted canola meals corresponds in the present study to a RSM toasted for 20 min. In a previous study [2], increasing the toasting time from 0 to 93 min decreased the lysine content by 23%, which is similar to the reduction in lysine content observed in the present study after 100 min of toasting, regardless of the initial contents.

Conversion of fructoselysine during 6 M HCl hydrolysis yields furosine (32%), pyridosine (16%) and regenerated lysine (56%) [24]. The *N* ^ε^-carboxymethyl-lysine (Fig. 4a), furosine (Fig. 4c) and *N* ^ε^-carboxyethyl-lysine (Fig. 4d) contents in the RSM linearly (*P* < 0.001) increased with increasing toasting time. The formation of these compounds with increasing toasting time does not completely account for the reduction in the lysine content, which also decreases linearly (*P* < 0.001; Fig. 4e). Whereas the lysine content decreases 15.7 μg/mg CP from the 0 min to the 120 min-toasted RSM, the sum of fructoselysine (calculated from furosine), *N* ^ε^-carboxymethyl-lysine and *N* ^ε^-carboxyethyl-lysine only increases 11.1 μg/mg CP in the same toasting time range. Other Maillard-derived compounds (e.g. [5-hydroxymethyl]-2-furfural) that were not determined in this experiment were probably also formed during toasting, which could account for this difference. The content of lysinoalanine (Fig. 4b) decreased during the initial 20 min of toasting, but remained constant with increasing toasting times. Formation of lysinoalanine is favored with an alkaline pH [25], which is unlikely to have been applied during toasting of the RSM. No lanthionine could be detected in any of the samples.

Most of the chemically modified compounds formed were present in the insoluble protein fraction. The contents of furosine (Fig. 4c) and *N* ^ε^-carboxyethyl-lysine (Fig. 4d) increased linearly (*P* < 0.001) with increasing toasting times, whereas there were linear (*P* = 0.006) and quadratic (*P* = 0.009) effects of toasting time on the *N* ^ε^-carboxymethyl-lysine (Fig. 4a) content. The content of lysine (Fig. 4e) decreased linearly (*P* < 0.001) with increasing toasting time. Contents (μg/mg CP) of furosine, *N* ^ε^-carboxymethyl-lysine, *N* ^ε^-carboxyethyl-lysine and lysine were higher in the insoluble protein fractions compared with the complete RSM. The removal of the soluble protein fraction probably concentrates the aggregated and chemically modified insoluble fraction. The presence of these Maillard-derived compounds in the insoluble fraction could delay the enzymatic cleavage of the available peptide bonds due to steric hindrance [26]. In addition to protein aggregation, as described above, the presence of these chemically modified compounds in the insoluble fraction could also explain the observed reduction of the *k* of the insoluble protein fractions. There were no indications of other chemical crosslinks occurring: lysinoalanine (Fig. 4b) contents in the insoluble fraction do not change with increasing toasting time, whereas no lanthionine could be detected in any of the samples.

### Molecular Size Distribution

**Fig. 5:**
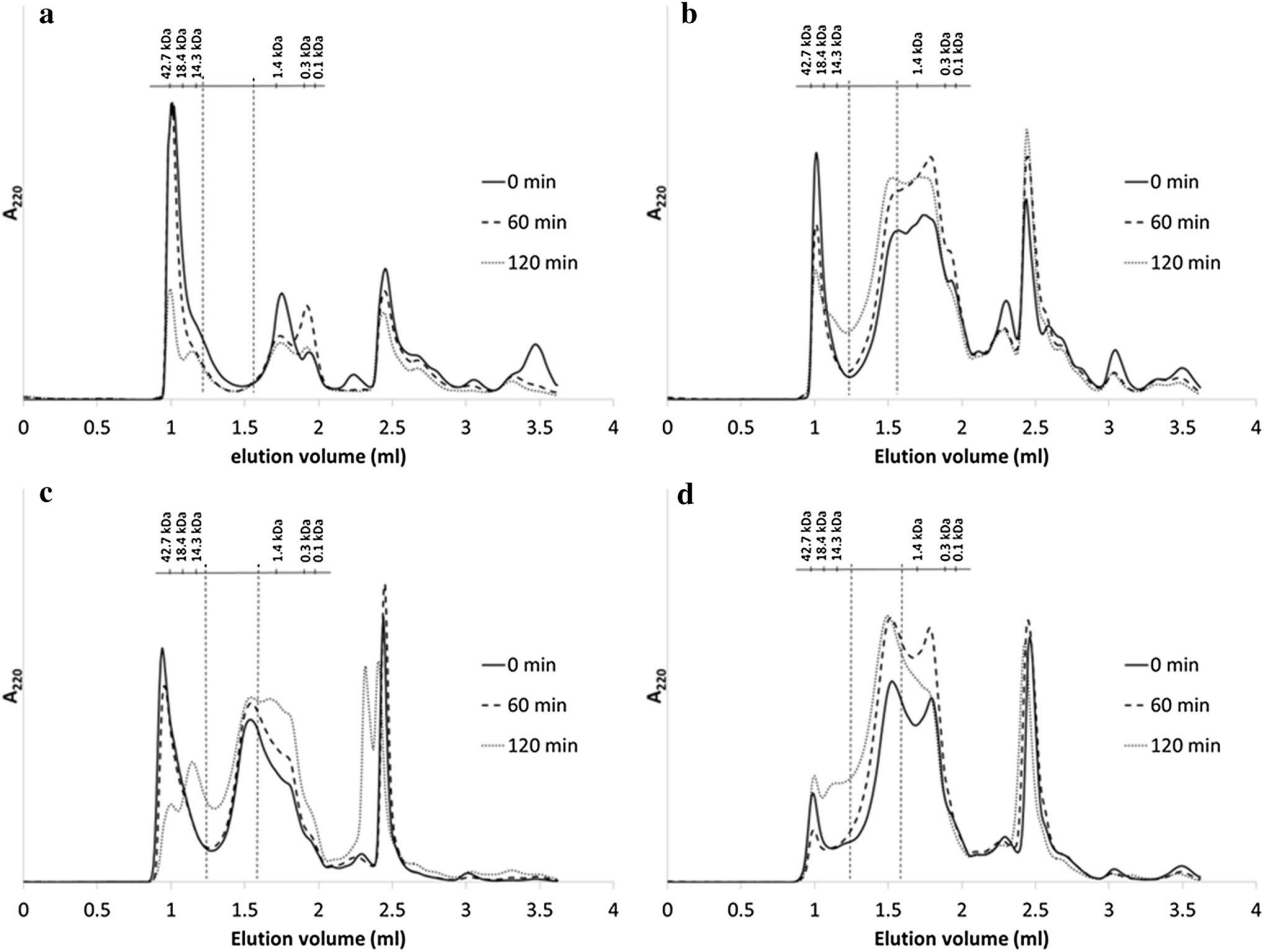
Size exclusion chromatograms of the **a** soluble fractions of rapeseed meals toasted for different times before hydrolysis and the hydrolysates of **b** rapeseed meals, **c** soluble and **d** insoluble protein fractions of the rapeseed meals toasted for different times. *Vertical dashed lines* represent the cut-off points of 10 and 1.5 kDa

Toasting time influenced the molecular weight distribution of the proteins in solution before hydrolysis (Fig. 5a) and the peptides obtained after hydrolysis of the RSM (Fig. 5b), the soluble (Fig. 5c) and the insoluble (Fig. 5d) protein fractions. The fractions in the chromatograms that elute after 2 ml consist of unspecified protein/peptides that bind to the column.

The largest fraction of soluble proteins from the intact RSM elute at a molecular mass >10 kDa (Fig. 5a). Intact soluble proteins and soluble protein aggregates can be located in this region [27]. The proportion of material >10 kDa decreased (linear *P* < 0.001, quadratic *P* = 0.04) with increasing toasting time (Table 3). This was also reflected by the linear increase (*P* < 0.001) of the proportion of proteins/peptides <10 kDa with increasing toasting time. This increase was probably the result of a higher relative representation of this highly soluble fraction compared with the decreasing contents of soluble intact proteins at longer toasting times.

**Table 3:** Relative molecular weight distribution (%) of intact RSM toasted for different times and the hydrolysates of these RSM, and soluble and insoluble protein fractions separated from these RSM

Toasting time	Intact RSM			Hydrolysates								
				RSM			Soluble protein fraction			Insoluble protein fraction		
	>10 kDa	10–1.5 kDa	<1.5 kDa	>10 kDa	10–1.5 kDa	<1.5 kDa	>10 kDa	10–1.5 kDa	<1.5 kDa	>10 kDa	10–1.5 kDa	<1.5 kDa
0 min	52.3	12.8	34.9	19.2	21.4	59.5	33.6	26.4	40.0	11.7	32.0	56.3
20 min	50.9	9.0	40.1	13.1	24.3	62.6	27.8	27.8	44.4	7.0	35.8	57.2
40 min	49.5	10.1	40.5	12.7	25.7	61.6	24.9	28.1	47.0	7.5	36.6	56.0
60 min	48.6	9.8	41.6	13.3	28.6	58.1	22.8	30.4	46.8	9.1	37.3	53.6
80 min	45.2	10.3	44.5	12.6	28.7	58.7	20.3	30.6	49.1	11.2	38.3	50.5
100 min	42.4	11.3	46.3	13.7	30.7	55.6	19.2	31.3	49.5	13.1	39.3	47.6
120 min	38.3	12.4	49.3	14.0	30.5	55.5	18.8	30.7	50.5	15.6	39.6	44.7
SEM	1.4	0.4	1.2	0.5	0.9	0.8	1.3	0.5	0.9	0.9	0.6	1.3
*P* value												
Linear	<0.001	0.15	<0.001^c^	0.34	<0.001^e^	<0.001^f^	<0.001	0.002^h^	<0.001^i^	0.003	<0.001	<0.001
Quadratic	0.04^a^	0.006^b^	0.76	0.006^d^	0.13	0.32	0.005^g^	0.18	0.07	<0.001^j^	0.02^k^	0.002^l^

Abbreviations: RSM, rapeseed meal; SEM, standard error of the mean

^a^Intact >10 kDa = 51.84 − 0.017 × time − 0.00,079 × time^2^ (*R* ^2^ = 0.91)

^b^intact 10–1.5 kDa = 11.45 − 0.07 × time + 0.00066 × time^2^ (*R* ^2^ = 0.63)

^c^Intact <1.5 kDa = 36.38 + 0.10 × time (*R* ^2^ = 0.85)

^d^RSM >10 kDa = 16.97 − 0.13 × time + 0.00093 × time^2^ (*R* ^2^ = 0.58)

^e^RSM 10–1.5 kDa = 22.83 + 0.073 × time (*R* ^2^ = 0.76)

^f^RSM <1 kDa = 62.63 − 0.060 × time (*R* ^2^ = 0.67)

^g^Soluble >10 kDa = 32.81 − 0.23 × time + 0.00099 × time^2^ (*R* ^2^ = 0.93)

^h^Soluble 10–1.5 kDa = 27.09 + 0.038 × time (*R* ^2^ = 0.60)

^i^Soluble <1 kDa = 42.68 + 0.071 × time (*R* ^2^ = 0.77)

^j^Insoluble >10 kDa = 10.10 − 0.093 × time + 0.0012 × time^2^ (*R* ^2^ = 0.91)

^k^Insoluble 10–1.5 kDa = 32.74 + 0.11 × time − 0.00041 × time^2^ (*R* ^2^ = 0.92)

^l^Insoluble <1 kDa = 57.16 − 0.014 × time − 0.00078  × time^2^ (*R* ^2^ = 0.97)

Hydrolysis of the RSM and their soluble and insoluble fractions changed their elution profiles compared with the soluble intact RSM, as higher proportions of material can be determined at lower molecular masses (<10 kDa; Table 3). In the hydrolysates of RSM, the proportion of material <10 kDa corresponded to 80–86% of the quantified area (i.e. excluding the a-specific binding, retention volume higher than 2 ml). The proportion of peptides >10 kDa in the hydrolysates of the RSM decreased after the initial 20 min of toasting and was not largely affected by a further increase in toasting time. Increasing the toasting time increased the overall molecular weight of these hydrolysates, as an increasing (linear, *P* < 0.001) proportion of peptides 1.5–10 kDa and a decreasing (linear, *P* < 0.001) proportion of peptides <1.5 kDa were determined at higher toasting times compared with lower ones. There was a negative correlation (*r* = −0.73, *P* < 0.01) between the proportion of peptides 1.5–10 kDa and the *k* of hydrolysis of the RSM, whereas a positive correlation (*r* = 0.84, *P* < 0.001) was determined for the proportion of peptides <1.5 kDa (Table 4). The proportion of peptides 1.5–10 kDa in the RSM hydrolysates was also positively correlated (*r* = 0.56, *P* < 0.05) to the DH_max_, whereas a negative correlation (*r* = −0.74, *P* < 0.01) was determined between the proportion of peptides <1.5 kDa and the DH_max_.

**Table 4:** Pearson correlation coefficients between the proportion of peptides after hydrolysis and the maximum degree of hydrolysis (DH_max_) and rate of hydrolysis (*k*) of the RSM and the soluble and insoluble protein fractions separated from these RSM

Proportion of peptides after hydrolysis	RSM		Soluble protein fraction		Insoluble protein fraction	
	DH_max_	*k*	DH_max_	*k*	DH_max_	*k*
>10 kDa	0.16	−0.01	−0.25	−0.44	0.02	−0.77**
1.5–10 kDa	0.56*	−0.73**	0.08	0.63*	0.44	−0.74**
<1.5 kDa	−0.74**	0.84***	0.32	0.25	−0.23	0.87***

*DH*_*max*_ maximum degree of hydrolysis, *k* rate of protein hydrolysis, *RSM* rapeseed meal

Significance level: * *P* < 0.05, ** *P* < 0.01, *** *P* < 0.001

The hydrolysates of the soluble protein fraction contained a larger proportion of material >10 kDa compared with the RSM hydrolysates (Table 3). This can be explained by the presence of intact soluble proteins, which were detected by SDS–PAGE in the hydrolysates of the RSM and the soluble protein fraction (results not shown). However, increasing the toasting time decreased (linear *P* < 0.001, quadratic *P* = 0.005) the proportion of material >10 kDa (Table 3). The nitrogen concentration before hydrolysis was similar for all the hydrolyzed soluble fractions. Therefore, the decrease in the proportion of material >10 kDa is not expected to be due to the decrease in solubility with increasing toasting times (as explained previously for the soluble intact RSM), but to the facilitated hydrolysis of soluble intact proteins. This was also reflected by the overall decrease in the molecular weight of the hydrolysates, as the proportion of peptides 1.5–10 kDa (*P* = 0.002) and <1.5 kDa (*P* < 0.001) linearly increased with increasing toasting times. The increase in the proportion of peptides <1.5 kDa with increasing toasting time obtained after hydrolysis of the soluble protein fraction can be explained by an increased denaturation of the soluble proteins, exposing cleavage sites that were initially not accessible for the enzymes [28]. A positive correlation (*r* = 0.63, *P* < 0.05) was determined between the proportion of peptides 1.5–10 kDa and the *k* of hydrolysis of the soluble protein fraction (Table 4).

In contrast to what was observed in the hydrolysates of the soluble protein fraction, increasing the toasting time increased the overall molecular weight of the peptides present in the hydrolysates of the insoluble protein fraction (Fig. 5d). The proportion of material >10 kDa decreased after the initial 20 min of toasting, but steadily increased with increasing toasting time (Table 3). This is not expected to be due to the presence of intact proteins, as no clear bands were detected by SDS–PAGE in these hydrolysates (results not shown). The proportion of material >10 kDa was negatively correlated to the *k* of hydrolysis (*r* = −0.77, *P* < 0.01) of the insoluble protein fraction (Table 4). Furthermore, increasing the toasting time increased (linear *P* < 0.001, quadratic *P* = 0.02) the proportion of peptides 1.5–10 kDa and decreased (linear *P* < 0.001, quadratic *P* = 0.002) the proportion of peptides <1.5 kDa. There was a negative correlation (*r* = −0.74, *P* < 0.01) between the proportion of peptides 1.5–10 kDa and the *k* of hydrolysis of the insoluble protein fraction, whereas a positive correlation (*r* = 0.87, *P* < 0.001) was determined with the proportion of peptides <1.5 kDa (Table 4).

The correlations between *k* and the size distribution of the peptides in the hydrolysates of RSM and the insoluble protein fraction could be explained by a shift of the hydrolytic mechanism from a more one-by-one type to a more zipper-type-dominated system with increasing toasting time [19, 29]. Hydrolysis of most proteins shows an intermediate behavior between these two types of hydrolytic mechanisms [19]. In the one-by-one type of hydrolysis, the cleavage of peptide bonds in one protein is followed by a fast cleavage into smaller peptides. Overall, a higher proportion of intermediate peptides (Table 3) was determined in this study at short toasting times compared with long toasting times. In contrast, in the zipper-type hydrolysis, several peptide bonds are cleaved simultaneously, which is followed by a slow conversion of large peptides into smaller peptides. The shift towards a zipper-type enzymatic cleavage renders peptides of larger sizes, compared with the more one-by-one type of hydrolysis. Hydrolysates that contain peptides with larger sizes, in addition to intact proteins, can probably be considered to be less digestible compared with those with smaller sizes.

## Conclusion

The rate of protein hydrolysis of the soluble protein fraction was 3–9-fold greater than that of the insoluble protein fraction. The decrease in the rate of hydrolysis of the RSM observed with increasing toasting time results from a combination of (1) the reduction in the proportion of fast hydrolysable soluble proteins to slowly digestible insoluble proteins and (2) the decrease in the rate of hydrolysis of the insoluble proteins with increasing toasting time due to the formation of disulfide bonds and/or chemically modified amino acid residues. In addition, increasing the toasting time results in an overall increase of the size of the peptides after hydrolysis. Positive correlations were obtained between the rates of protein hydrolysis of the RSM and the insoluble protein fraction with the proportion of small peptides (<1.5 kDa) after hydrolysis.

## Abbreviations

DH_max_: Maximum degree of hydrolysis
DTT: Dithiothreitol
*k*: Rate of hydrolysis
RSM: Rapeseed meal
